# Environmental, social, and corporate governance activities with employee psychological well-being improvement

**DOI:** 10.1186/s12889-021-12350-y

**Published:** 2022-01-06

**Authors:** Xiangdan Piao, Jun Xie, Shunsuke Managi

**Affiliations:** 1 Urban Institute & Faculty of Humanities and Social Science, Iwate University, 3-18-34 Ueda, Morioka, Iwate 020-8550 Japan; 2 Urban Institute & Department of Civil Engineering, Kyushu University, 744 Motooka Nishi-ku, Fukuoka, 819-0395 Japan

**Keywords:** Environmental, social, and corporate governance, Longitudinal data, Japan, Occupational stress

## Abstract

**Background:**

Environmental, social, and governance (ESG) engagement is expected to benefit corporations in terms of their efficiency and sustainability. The transformative change in management practices would not only provide support for employees but also bring about additional workload, which may affect employee psychological well-being. However, the examination of the relationship between corporate ESG activities and occupational stress is scarce; hence, this study aims to fill this knowledge gap.

**Methods:**

In total, 110,351 observations were collected from 41,998 employees regarding occupational stress to reflect employee psychological well-being. The data were derived from 11 corporations in Japan from 2017 to 2019. Data on ESG activities were collected from the MSCI ESG database from 2015 to 2017. The effect of 1-year lagged corporate ESG activities on employee psychological well-being was investigated using a lagged variable linear regression model.

**Results:**

Positive and negative relationships were found between corporate environmental activities and occupational stress. Activities that reduce water stress during operation and adopt clean technology were found to benefit employees’ psychological well-being. On the contrary, the program for reducing toxic emissions and waste lowered employees’ occupational stress levels significantly. Regarding corporate social activities, the improvement of job satisfaction or work-life balance was associated with occupational stress. However, corporate governance activities were found to have unfavorable effects on employees’ psychological well-being.

**Conclusion:**

The effects of corporate ESG activities on employees’ psychological well-being are found. The managerial implications suggest that caring for employees’ occupational stress during the implementation of environmental activities is necessary, and the adoption of social activities could enhance employees’ psychological well-being. Notably, corporate governance activities are a stressor for employees; top management teams should pay attention to it.

## Background

Over the past several decades, a growing number of corporations began to incorporate environmental, social, and governance (ESG) activities into their management practices, following the stakeholder theory that aims to enhance corporate sustainability [1]. Previous studies noted that ESG activities could have a favorable relationship with corporate efficiency and sustainability [2, 3]. To be specific, Xie et al. [2] found that corporate transparency regarding ESG information could strengthen corporate efficiency. Similarly, Alsayegh et al. [3] concluded that corporate environmental and social disclosures are positively associated with corporate performance. Although the evidence showed the benefits of ESG activities on firm performance, Drempetic et al. [4] pointed out that a firm size bias exists in the current measurement of the ESG ratings, which might not provide an exact evaluation of the corporate sustainability performance that the responsible investors and related stakeholders need. Furthermore, numerous studies found that mixed relationships exist between corporate sustainability performance and financial performance by using the current ESG ratings [5–7], which also implies that it is necessary to consider factors that may detriment corporate performance during the implementation of ESG activities.

Occupational stress is a vital factor for determining corporate performance and also a key issue regarding employee well-being within the scope of corporate sustainability. Prior studies suggested that poor employee psychological well-being is associated with lower performance and increased turnover rate [8–12]. For example, Kazmi et al. [11] investigated occupational stress and job performance of Canadian blue-collar and managerial workers. They found a strong negative relationship between the stated variables, which indicated that higher levels of occupational stress resulted in worse job performance. Consistent results have also been reported in Japanese firms [13]. As ESG engagement becomes stronger, different workloads and job support may affect employees’ occupational stress [14]. However, occupational stress is neglected in the measurement of ESG ratings, and studies on the relationship between corporate ESG activities and employee psychological well-being are limited.

To improve employees’ psychological well-being, scholars have recently focused on theoretical and empirical investigation of employees’ occupational stress and workplace environment. When an insufficient workplace environment supports these workplace characters, it causes depression, sleeplessness, and anxiety among employees. Occupational stress is also associated with mental illness [15–18].

Theoretically, the job-demand-control model or job-demand-resource model has been developed based on previous literature on occupational stress [19–21*]*. The job-demand-control model, proposed by Schnall et al. [19] in 1990s, provides a theoretical framework for examining the relationship between workplace and occupational stress. This model considers that great job demand with low job control increases employees’ occupational stress, which is associated with a higher risk of mental illness. The job demand includes an increased workload or high quality, whereas job control is associated with job control authority on skill use and working process. The job-demand-resource model, developed in the 2000s suggests high job demand and low resources for employees, worsen their occupational stress [20, 21]. Low resources include poor support from family, boss, and colleagues. Similarly, in the framework of theory in organizational management, the stakeholder theory describes that corporations’ activities affect business entities like employees directly or indirectly [22, 23]. The models are applied to enhance the safe and comfortable working environment, well-organized working conditions, employees’ health and safety and achievement of employees’ work-life balance.

In line with the above concepts and stress models, empirical evidence has been accumulated in the recent past. Along with the job-strain model, the work environment characteristics of social support, effort-reward balance, work-life balance, job security, and harassment appear to have significant effects on employees’ psychological well-being [24–42]. When studies point out corporations’ ESG engagement, it is expected to benefit corporations in terms of their efficiency and sustainability [1–3]. Evidence ensures that corporation’s sustainability and employees’ well-being is expected to provide insightful evidence, which is shown in this study.

This study builds on the literature on the following aspects. Against this backdrop, this study employs an empirical examination of the effects of corporate ESG behavior on employees’ psychological well-being based on a large sample of the occupational stress survey and corporate ESG database. The results clarify the impacts of the key ESG activities on employees’ occupational stress. This study contributes to the existing literature in several aspects. First, previous studies on occupational stress usually focused on social activities that are thought to be able to provide a better workplace for employees, while seldom discussing the impacts of environmental, and governance activities on occupational stress. To our knowledge, this is the first attempt to examine the impacts of both social, and environmental and governance activities on occupational stress. Second, reverse causality issues are considered in this study, which can provide more robust results on the impact of ESG activities. Third, the detailed evaluations of ESG activities allow us to separate the effects of vital activities in each aspect of ESG activities, particularly the complexity of environmental activities. These results are expected to provide evidence to improve the measurement of ESG ratings by mapping the mixed relationships between corporate ESG activities and employee psychological well-being. Moreover, it helps deepen the knowledge of building a sustainable society through solutions at the corporate level.

The remainder of this paper is organized as follows. Materials and methods Section presents the data and the variable settings. Data Section presents the methodology, and Occupational stress-29 Section summarizes the empirical results. Finally, Environmental, social, and corporate governance activities Section presents the conclusions.

## Materials and methods

### Data

To examine the relationship between corporate ESG activities and employee’s psychological well-being, this study used large-scale occupational stress data from 2017 to 2019 (panel data) provided by a third-party company and corporate ESG data from 2015 to 2019.

Large-scale employee longitudinal data collected occupational stress levels from 11 corporations in Japan. These companies are responsible for checking the employee’s psychological well-being annually based on the Ministry of Health, Labor, and Welfare guidelines [43]. The occupational stress data records included data for all the respondents. In total, 110,351 observations were collected from 2017 to 2019, including 31,871 observations in 2017, 36,482 observations in 2018, and 41,998 observations in 2019. The study design was approved by the appropriate legal and ethics review board. The employees were provided with informed consent according to legal and ethical guidelines. These data do not target personal health information, and personal information is non-identifiable, and the process of research has proceeded in accordance with ethical guidelines. Regarding corporate ESG activities, the MSCI conducted an original assessment of corporate ESG activities and scored them to identify the corporate ESG risks and their relative management ability for enhancing resilience [44].

#### Occupational stress-29

Employee psychological well-being and the occupational stress-29 score were collected from 11 corporations through a brief occupational stress questionnaire from the Japanese Ministry of Health, Labor and Welfare by a third-party company. The Japanese government provided a standard guideline for corporations to investigate occupational stress levels [43]. The Occupational-stress-29 scale, guided by the Japanese government, includes 29 items to measure psychological well-being.

The following are questions regarding how you felt during the past month. Please choose the most applicable answer. (1) I feel energetic; (2) I feel cheerful; (3) I feel lively; (4) I feel angry; (5) I feel frustrated; (6) I feel irritated; (7) I feel exhausted; (8) I feel completely worn out; (9) I feel lethargic; (10) I feel tense; (11) I feel anxious; (12) I feel restless; (13) I feel depressed; (14) I don’t feel like doing anything; (15) I cannot focus on things; (16) I feel blue; (17) I cannot concentrate on my job; (18) I feel sad; (19) I feel dizzy; (20) My body aches; (21) I have headaches; (22) My neck and shoulders are tense; (23) My back hurts; (24) I get eyestrain; (25) I experience shortness of breath or excessive heartbeats; (27) I have an upset stomach; (28) I don’t feel hungry; (29) I have constipation or diarrhea; I cannot sleep well. Each question among occupational stress items had the same answer choices: almost never = 1, sometimes = 2, often =3, and almost always = 4. The occupational stress-29 score is the unweighted summation of the valued number choice of 29 questions, ranging from 29 to 116. Higher scores indicated worse psychological well-being. Notably, the first three items on the scale are reverse scored. The occupational stress scale is stated in Table 1.

**Table 1:** Employees’ occupational stress in Japan

Variable	Mean	Std. Dev.	Min	Max
Occupational stress-29 score	56.3	14.1	29	116
Employee age	43.4	11.6	15	75
Female dummy	0.2	0.4	0	1
Firm size	9.9	0.9	8.2	12.3
Leverage	144.1	72.5	70.8	324.0

Note: The Occupational stress-29 score ranged between 29 and 116, indicating greater value shows the worse occupational stress level. Data source: Large-scale occupational stress data from 2017 to 2019 (panel data) is provided by PEACEMIND Inc. and corporate data is collected from Refinitiv Eikon

#### Environmental, social, and corporate governance activities

The MSCI ESG Database provides a set of ESG scores to evaluate the corporate capacity for dealing with various kinds of ESG key issues when facing corresponding opportunities or risks. In order to test how ESG activities are related to employee’s occupational stress, we consider the ESG Pillar Scores, as well as related Key Issue Scores and Management Scores as indices of ESG activities, including 8 environmental indices, 5 social indices and 6 corporate governance indices. The detailed description of each index is shown in Table 2. Here, Pillar Scores across three ESG pillars are calculated based on the weighted average of all key issue scores underlying each pillar, ranging from 0 to 10, in which a larger number indicates a larger capacity in the corresponding pillar. Key issue scores indicate the corporate’s capacity for a certain ESG issue that can be either risks or opportunities. The key issue scores also range from 0 to 10, which are adjusted to the degree of risks or opportunities that a firm may face given the level of their corresponding management practice. The management scores, ranging from 0 to 10, indicate a company’s management practices, including strategies, programs, and proven track records on certain key issues. It is worth noting that for a firm with a low level of management (management score less than 5), higher opportunities in certain ESG issues will lead to lower key issue scores. On the contrary, if a firm has a high level of management (management score larger than 5), the key issue scores will be positively related to the degree of ESG opportunity. The final key issue scores are negatively related to the degree of risk exposure while positively related to the management score. Thus, the management score is a vital indicator when determining a firm’s capacity to deal with ESG issues. However, the high level of management in ESG issues would also bring about concerns on employees’ occupational stress, which may decrease the employees’ well-being and finally detriment the firm outcomes. Thus, we test both the key issue score and management score for the investigated ESG activities in this study.

**Table 2:**
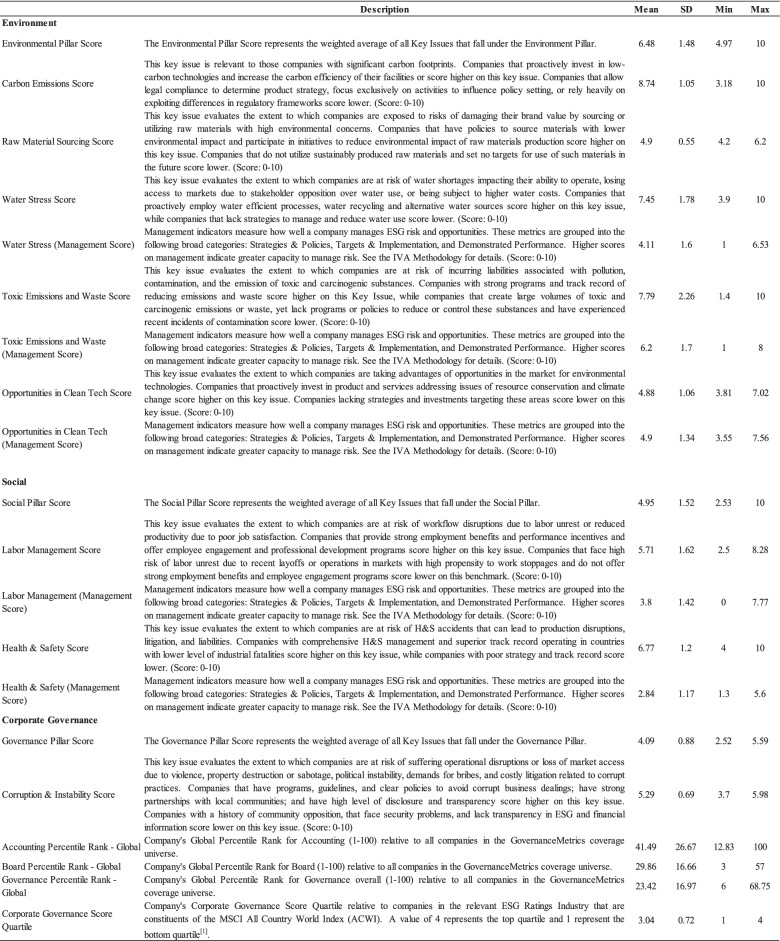
Descriptions of ESG indices

Notes: Variable descriptions are derived from MSCI ESG Database. The reversed transformation of corporate governance score quartile from MSCI is utilized, in which 4 indicates the top quartile and 1 is the bottom quartile

As for environmental aspects, we focus on the key issues of carbon emissions, raw material sourcing, water stress, toxic emissions and waste, and clean technology, which are all essential environmental issues that attract attention from the public. In terms of the social aspect, we investigate the key issues of labor management and health and safety issues directly associated with employees’ well-being. Regarding the corporate governance aspect, key issues of corruption and instability, accounting, board, and corporate governance scores were used to test the impacts of governance activities on occupational stress.

### Methodology

The following estimation was used to explore the effect of corporate ESG performance on employees’ psychological well-being.

1 $${s}_{ijt}={\beta}_0+{\beta}_1{W}_{j,t-1}+{\beta}_2{D}_t+{\beta}_3{Y}_{jt}+{\beta}_4{X}_{ijt}+{u}_i+{\varepsilon}_{ijt}$$

where *s*_*ijt*_ is the occupational stress-29 score, ranging from 29 to 116, of employee *i* in corporation *j* in year *t*. A greater number indicates higher stress levels, and corresponds to employees’ poor psychological well-being. In Eq. (1), *W*_*j*, *t* − 1_ denotes the indices of the corporation’senvironmental, social, and governance activities in the year *t* − 1.. Corporate activities were estimated separately. The reverse effect of employees’ occupational stress on corporations’ ESG determinants might be mixed; the 1-year-lagged ESG activities are adopted to remove these reverse effects [41, 44]. *D*_*t*_ denotes the year dummy variable, and *Y*_*jt*_ represents the corporation characteristics, firm size and leverage, which is thought to affect both corporate ESG activities and employee psychological well-being. *X* is a vector of covariates including employee age and female dummy variable. *u*_*i*_ denotes the time-invariant of employee’s attributes and *ε*_*ijt*_ denotes the error term. *β*_0_, *β*_1_, *β*_2_, *β*_3_ and *β*_4_ are the estimated parameters.

The parameter *β*_1_ captures the effects of corporate environmental, social, and governance activities. If the estimated parameters are negative, it suggests that the improvement of corporate ESG activities has a positive impact on employees’ psychological well-being. This study aimed to fill the knowledge gap by examining the effect of corporations’ environmental, social, and governance performance on employees’ psychological well-being based on the lagged variable OLS model, which is adopted by previous studies [45–48].

The coefficients of labor management, labor management (management), health & safety and health & safety (management) are negative values and statistically significant. The results indicate that job-demand-control model is supported. Better work environment with low job demand, high job-control and more support from surrounding people have favorable effects on employees’ occupational stress. Similarly, the coefficients of corporations’ environment, social and governance activities are positive/negative and statistically significant. It suggests the corporations’ ESG activities significantly impact the employees’ occupational stress, which suggests the confirmation of the stakeholder theory.

## Results

Tables 3, 4, and 5 present the regression results of Eq. (1) to show the relationship between detailed corporate ESG activities and occupational stress using the lagged variable model. The dependent variable was occupational stress, which revealed psychological well-being. Results ranged from 29 to 116, with a greater number indicating poor psychological well-being. Here, coefficients of negative value indicate favorable impacts on employees’ psychological well-being. Each index of corporate ESG activity was estimated separately.

**Table 3:**
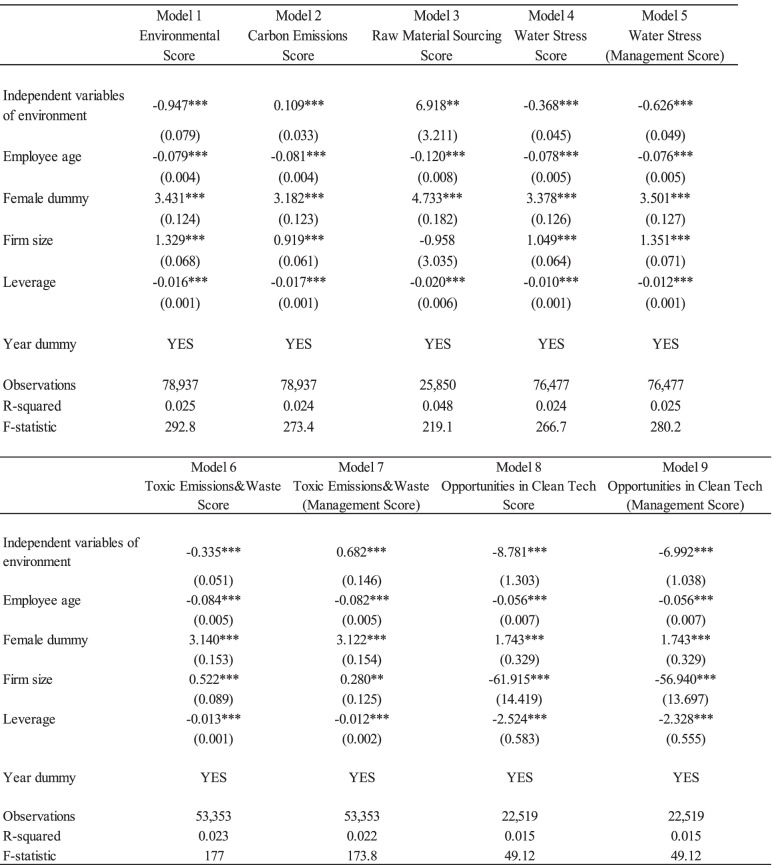
Effects of corporate environmental activities on the occupational stress

Note: Standard errors are in parentheses. *** *p* < 0.01, ** *p* < 0.05, * *p* < 0.1. The lag-variable model (LV1) was used from Model 1 to Model 9. The independent variables of the environment are Environmental Score; Carbon Emissions Score; Raw Material Sourcing Score; Water Stress Score; Water Stress (Management Score); Toxic Emissions & Waste Score; Toxic Emissions & Waste (Management Score); Opportunities in Clean Tech Score, and Opportunities in Clean Tech (Management Score). The sample size is smaller than the number of total observation is because the missing values of independent variables

**Table 4:**
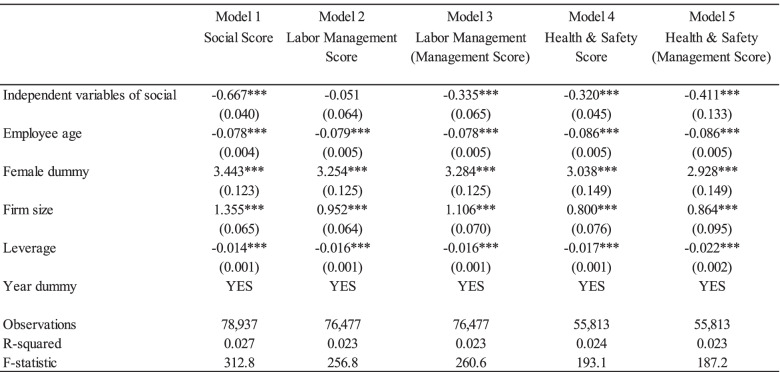
Effects of corporate social activities on the occupational stress

Note: Standard errors are in parentheses. *** *p* < 0.01, ** *p* < 0.05, * *p* < 0.1. The independent variables of social are Social Score; Labor Management Score; Labor Management (Management Score); Health & Safety Score, and Health & Safety (Management Score). Among other models, the lag-variable model (LV1) is selected. The sample size is smaller than the number of total observation is because the missing values of independent variables

**Table 5:**
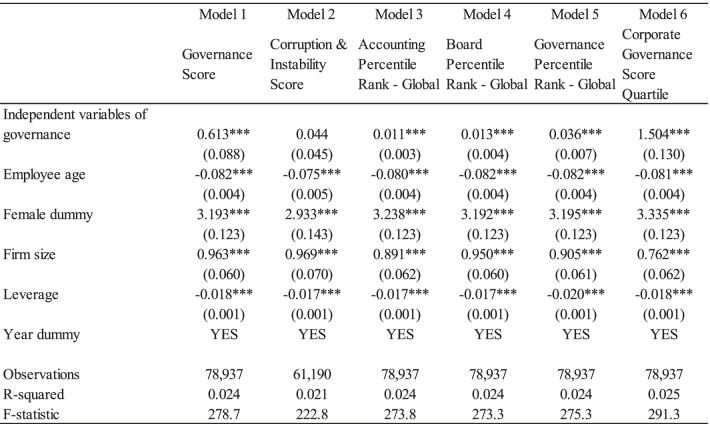
Effects of corporate governance activities on the occupational stress

Note: Standard errors are in parentheses. *** *p* < 0.01, ** *p* < 0.05, * *p* < 0.1. The independent variables of governance are Governance Score; Corruption & Instability Score; Accounting Percentile Rank—Global; Board Percentile Rank—Global; Governance Percentile Rank—Global, and Corporate Governance Score Quartile. The lag-variable model (LV1) is used from among the models. The reversed corporate governance score quartile is utilized, where 4 indicates the top quartile and 1 indicates the bottom quartile. The sample size is smaller than the number of total observation is because the missing values of independent variables

Table 3 illustrates the effects of corporate environmental activities on employees’ psychological well-being. First, the environmental pillar score presented positive impact on employees’ occupational stress. Specifically, the coefficients of water stress (Models 4 and 5) were negative values, and were statistically significant, showing favorite effects on employees’ occupational stress. In addition, the coefficients of opportunities in clean tech scores (Model 8) and opportunities in clean tech management scores (Model 9) are negative and statistically significant at the 1% level. However, environmental activities regarding carbon emission (Model 2) and raw material sourcing (Model 3) and Management Score of Toxic Emissions & Waste (Model 7) presented positive coefficients. They were statistically significant, indicating that they had negative effects on employees’ occupational stress.

Table 4 presents the effects of corporate social activities and employee psychological well-being. Similar to the results in the environmental pillar score, Model 1 show that the social pillar score has a significant and positive impact on employees’ occupational stress levels. The coefficients of Labor Management (Management Score); Health & Safety Score; Health & Safety (Management Score) are negative and statistically significant at 1%. The coefficient of the Labor Management score was negative but statistically insignificant (Model 2). Table 5 illustrates the significant effects of corporate governance activities on employees’ psychological well-being. In contrast to the environmental and social pillar scores, the coefficient of corporate governance pillar score is positive and significant, implying negative impacts on employees’ occupational stress. The coefficients of the Accounting Percentile Rank—Global; Board Percentile Rank—Global; Governance Percentile Rank—Global, and Corporate Governance Score Quartile were positive and statistically significant at the 1% level. Finally, the control variables, including personal-specific and firm-specific characteristics, were found to have significant effects on employees’ occupational stress. The results are consistent across all the above models (see Table 6). The coefficients of employee age and firm’s financial leverage are negative and statistically significant at 1%. On the contrary, the coefficients of firm size and female dummy are positive and significant.

**Table 6:**
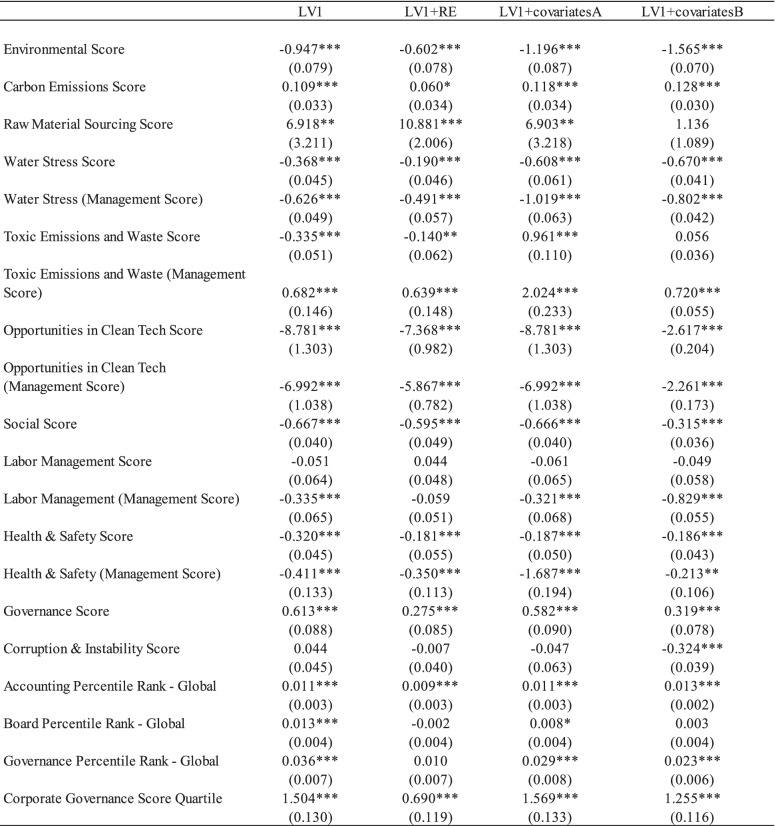
Impacts of corporates’ environmental, social, government activities on the occupational stress (robustness check)

Note: Standard errors are in parentheses. *** *p* < 0.01, ** *p* < 0.05, * *p* < 0.1. LV1 denotes the 1-year-lagged variable with the OLS model; LV1 + RE indicates the 1-year-lagged variable with the random effect model. Covariate A includes the employee age, female dummy, firm size, leverage year dummy, and industry dummy variables. Covariate B has a female dummy, firm size, leverage year dummy

## Discussion

Using the occupational stress data and corporation’s ESG activity data in Japan, we explore the effects of preventive corporate ESG activities on employees’ occupational stress. Both the ESG pillar scores and specific corporates’ activities present mixed relationships with occupational stress, when previous studies appear more attentions on within companies’ organizations, consistent results with the company’s social activities of ESG [18].

The results in this study build on previous literature on the following aspects. First, the environmental, social and governance pillar score presents significant relationships with occupational stress, implying that corporate environmental activities have significant impacts on emplyees’ occupational stress, which suggest that stakeholder theory is confirmed [22, 23]. However, the specific environmental activities presented mixed results. For example, corporations’ improved capacity for water-efficient processes, water recycling, and alternative water sources is found to benefit employees’ psychological well-being. The lack of strategies for water management may worsen the firm’s daily operations, resulting in higher employees’ occupational stress levels. Similarly, companies’ increased opportunities to use environmental technology on products and services could benefit employee’s psychological well-being. These results imply that sufficient working resources and strategies in the pursuit of future opportunities may enhance employees’ morale and alleviate occupational stress. On the contrary, companies that implemented strong programs to reduce pollution, contamination, and toxic emissions, carbon emissions, raw material sourcing could worse employees’ well-being. If the environmental activities require strong programs, dealing with these issues may harm employees’ health. Implementing such activities could increase the workload and health risk, which may worsen their psychological status [18, 21, 26, 36]. However, the significant level is relatively lower, and the management score of toxic emissions and waste presented non-significant results, which implies that it is possible to improve the implementation of toxic emissions and waste strategy while simultaneously caring for the employees’ psychological well-being.

Second, corporates’ social pillar score appears to have faverable effects on employees’ occupational stress, and mixed effects on occupational stress are confirmed in specific activities. In line with the previous studies, work environment is associated with improvement in employee psychological well-being [24–42]. The results suggest job-demand-control model and job-demand-resource model are supported that better work environment with low job demand, high job-control, and more support from surrounding people, have favorable effects on employees’ occupational stress. Employees’ psychological well-being in Japanese firms are affected by health and safety management. Improvement in corporates health and safety management is expected to release the employees’ occupational stress in Japanese firms. On the contrary, the positive effect of labor management on employees’ psychological well-being, because the well-organized labor management increases employee competition.

Third, company’s governance activities have unfavorable effects on occupational stress. Increasing the company’s global accounting rank had a negative effect on employees’ occupational stress levels. Similarly, improving the company’s board’s global rank and governance activities may worsen employees’ psychological well-being. The robustness results were observed from the relative quartile of the ESG rating industry among all countries. Additionally, the better corporate governance score quartile unfavorably influenced employees’ psychological well-being. Overall, these results indicate that corporate governance activities could be a stressor on employees in many aspects, including accounting, board, and the general requirements to enhance corporate governance. The procedures of strengthening corporate governance may increase the employees’ workload and pressures to comply, resulting in higher stress. For example, better work-reward ratio, reduce the work quantity for each employee to better the employee psychological well-being [23, 24, 37, 39], and these benefits might worsen the current corporates accounting.

In sum, the comprehensive ESG rating is a convenient tool to evaluate the firm’s capacity on ESG issues. However, it may ignore the potential unfavorable influence on daily operations and employees’ mental health. In our results, specific environmental activities have both positive and negative effects on occupational stress; where activities that bring about future benefits or support the daily operations have a positive effect on employees’ psychological well-being, while activities that are higher in health risks and require stronger programs could be a stressor for employees. The environmental activities that include a wide range of stakeholders, such as carbon emission and raw material sourcing, have negative effects on occupational stress. Corporate social activities and labor management programs that caters to employees’ job satisfaction provide strong employment benefits, incentives, and professional support, resulting in lower occupational stress. On the contrary, corporate governance activities negatively affect employees’ psychological well-being. The pursuit of improvement in corporate governance ranking, which requires complex and continuous work to meet the compliance and rating systems, may become a stressor for employees.

This study attempts to fill the void about the impacts of ESG activities on occupational stress. However, there are still limitations in this study. First, the ESG engagement is still in progress, and the lack of ESG information limited the number of observed firms. The employees’ demographic factors cannot be obtained because of the personal information protection policy. Fortunately, the large scale of observations made up for the deficiency of samples to some extent. Second, as per the methodology, the control variables in this study might not be enough. Hence, family structure and occupational status should be added as control variables. Unfortunately, there are limitations in the datasets as well. As the purpose of this survey targets employees’ occupational stress following the Japanese government’s guidelines, the socio-economic and demographic characteristics are omitted in the survey. These limitations indicate that further in-depth investigation is necessary based on relevant datasets.

## Conclusion

Growing numbers of companies have increased ESG engagement in their management strategy, which impacts ESG activities on occupational stress and requires in-depth investigations on the relationship between these activities and employee’s psychological well-being. This study confirmed the effects of corporate environmental, social, and governance activities on employees’ psychological well-being. Based on these results, several managerial implications can be summarized to enhance corporate sustainability. First, the positive and negative effects of environmental activities suggest that it is necessary to improve the implementation process by catering for employees’ occupational stress, especially when dealing with environmental issues of higher health risks and workload. Second, the result of the strong and significant impact of social activities encourages firms to adopt labor-management programs, enhancing employees’ job satisfaction and leading to higher productivity. Finally, the unfavorable impacts of corporate governance activities indicate that employees’ occupational stress is an urgent issue that needs to be considered when enhancing corporate governance and compliance strategies.

## Data Availability

The data is publicly unavailable, however, the data is accessible when PEACEMIND Inc. provides data access permission.

## Abbreviations

ESG: Environmental social and corporate governance
MSCI: Morgan Stanley Capital International

## References

[1] Freeman REE, McVea J (2001). A stakeholder approach to strategic management. SSRN Electron J.

[2] Xie J, Nozawa W, Yagi M, Fujii H, Managi S (2019). Do environmental, social, and governance activities improve corporate financial performance?. Bus Strateg Environ.

[3] Alsayegh MF, Abdul Rahman R, Homayoun S (2020). Corporate economic, environmental, and social sustainability performance transformation through ESG disclosure. Sustainability..

[4] Drempetic S, Klein C, Zwergel B (2020). The influence of firm size on the ESG score: corporate sustainability ratings under review. J Bus Ethics.

[5] Peloza J (2009). The challenge of measuring financial impacts from investments in corporate social performance. J Manage.

[6] Fujii H, Iwata K, Kaneko S, Managi S (2013). Corporate environmental and economic performance of Japanese manufacturing firms: empirical study for sustainable development. Bus Strateg Environ.

[7] Trumpp C, Guenther T (2017). Too little or too much? Exploring U-shaped relationships between corporate environmental performance and corporate financial performance. Bus Strateg Environ.

[8] Kachi Y, Inoue A, Eguchi H, Kawakami N, Shimazu A, Tsutsumi A (2020). Occupational stress and the risk of turnover: a large prospective cohort study of employees in Japan. BMC Public Health.

[9] Avey JB, Luthans F, Jensen SM (2009). Psychological capital: a positive resource for combating employee stress and turnover. Hum Resour Manag.

[10] Subha I, Shakil A (2009). Impact of stress on employee productivity, performance and turnover: an important managerial issue. Int Rev Bus Res Pap.

[11] Kazmi R, Amjad S, Khan D (2008). Occupational stress and its effect on job performance: a case study of medical house officers of district Abbottabad. J Ayub Med Coll Abbottabad.

[12] Muraale S, Basit A, Hassan Z (2017). Impact of job stress on employee performance. Int J Account Bus Manag.

[13] Ida H, Miura M, Komoda M, Yakura N, Mano T, Hamaguchi T (2009). Relationship between stress and performance in a Japanese nursing organization. Int J Health Care Qual Assur.

[14] Brough P, Drummond S, Biggs A (2018). Job support, coping, and control: assessment of simultaneous impacts within the occupational stress process. J Occup Health Psychol.

[15] Beehr TA, Newman JE (1978). Job stress, employee health, and organizational effectiveness: a facet analysis, model, and literature review. Pers Psychol.

[16] Caplan, RD, Sidney, C, John, FRP, Harrison, VR, Pinneau, SR. Job Demands and worker health: main effects and occupational differences. US Department of Health, education and welfare, public health service, Center for Disease Control, National Institute for Occupational Safety and Health, Washington, D.C. 1975.

[17] Margolis BL, Kroes WH, Quinn RP (1974). Job stress: an unlisted occupational hazard. J Occup Med.

[18] McGrath JE, Dunnette M (1976). Stress and behavior in organizations. Handbook of industrial and organization psychology.

[19] Schnall PL, Landsbergis PA, Baker D (1994). Job strain and cardiovascular disease. Annu Rev Public Health.

[20] Bakker AB, Demerouti E (2007). The job demands-resources model: state of the art. J Manag Psychol.

[21] Demerouti E, Bakker AB, Nachreiner F, Schaufeli WB (2001). The job demands-resources model of burnout. J Appl Psychol.

[22] Phillips R. Stakeholder theory and organizational ethics. Berrett-Koehler Publishers. 2003.

[23] Miles S (2012). Stakeholder: essentially contested or just confused?. J Bus Ethics.

[24] Aronsson G, Theorell T, Grape T, Hammarström A, Hogstedt C, Marteinsdottir I, Skoog I, Träskman-Bendz L, Hall C (2017). A systematic review including meta-analysis of work environment and burnout symptoms. BMC Public Health.

[25] Bhui, KS, Dinos, S, Stansfeld, SA, White, PD. A synthesis of the evidence for managing stress at work: a review of the reviews reporting on anxiety, depression, and absenteeism. J. Environ. Public Health. 2012, 515–874. 10.1155/2012/515874. 10.1155/2012/515874 PMC3306941 22496705

[26] Bonde JPE (2008). Psychosocial factors at work and risk of depression: a systematic review of the epidemiological evidence. Occup Environ Med.

[27] Einarsen S, Nielsen MB (2015). Workplace bullying as an antecedent of mental health problems: a five-year prospective and representative study. Int Arch Occup Environ Health.

[28] Finne LB, Knardahl S, Lau B (2011). Workplace bullying and mental distress—a prospective study of Norwegian employees. Scand J Work Environ Health.

[29] Ganster DC, Rosen CC (2013). Work stress and employee health: a multidisciplinary review. J Manag.

[30] Harvey SB, Modini M, Joyce S, Milligan-Saville JS, Tan L, Mykletun A, Bryant RA, Christensen H, Mitchell PB (2017). Can work make you mentally ill? A systematic meta-review of work-related risk factors for common mental health problems. Occup Environ Med.

[31] Hassard, J, Teoh, KRH., Visockaite, G, Dewe, P, Cox, T. The cost of work-related stress to society: a systematic review. J. Occup. Health Psychol. 2018;23:1–17. 10.1037/ocp0000069. 10.1037/ocp0000069 28358567

[32] Kouvonen, A, Oksanen, T, Vahtera, J, Stafford, M, Wilkinson, R, Schneider, J, Väänänen, A, Virtanen, M, Cox, SJ, Pentti, J, Elovainio, M, Kivimäki, M. Low workplace social capital as a predictor of depression: the Finnish public sector study. Am J Epidemiol 2018;167:1143–1151. https://doi.org/10.1093/aje/kwn067 10.1093/aje/kwn067 18413361

[33] Moen P, Kelly EL, Fan W, Lee SR, Almeida D, Kossek EE, Buxton OM (2016). Does a flexibility/support organizational initiative improve high-tech employees’ well-being? Evidence from the work, family, and health network. Am Social Rev.

[34] Nieuwenhuijsen, K, Bruinvels, D, Frings-Dresen, M. Psychosocial work environment and stress-related disorders, a systematic review. Occup Med 2010;60:277–286. https://doi.org/10.1093/occmed/kqq081 10.1093/occmed/kqq081 20511268

[35] Oksanen T, Kouvonen A, Kivimäki M, Pentti J, Virtanen M, Linna A, Vahtera J (2008). Social capital at work as a predictor of employee health: multilevel evidence from work units in Finland. Soc Sci Med.

[36] Pohling, R, Buruck, G, Jungbauer, KL, Leiter, MP. Work-related factors of presenteeism: the mediating role of mental and physical health. J Occup Health Psychol 2016;21:220–234. https://doi.org/10.1037/a0039670. 10.1037/a0039670 26322439

[37] Rugulies R, Aust B, Madsen IE (2017). Effort–reward imbalance at work and risk of depressive disorders. A systematic review and meta-analysis of prospective cohort studies. Scand J Work Environ Health.

[38] Stansfeld S, Candy B (2006). Psychosocial work environment and mental health—a meta-analytic review. Scand J Work Environ Health.

[39] Stansfeld SA, Shipley MJ, Head J, Fuhrer R (2012). Repeated job strain and the risk of depression: longitudinal analyses from the Whitehall II study. Am J Public Health.

[40] Too LS, Leach L, Butterworth P (2020). Is the association between poor job control and common mental disorder explained by general perceptions of control? Findings from an Australian longitudinal cohort. Scand J Work Environ Health.

[41] Umene-Nakano, W, Kato, TA, Kikuchi, S, Tateno, M, Fujisawa, D, Hoshuyama, T, Nakamura, J. Nationwide survey of work environment, work–life balance and burnout among psychiatrists in Japan. PLoS One 2013;8:e55189. https//doi.org/10.1371/journal.pone.0055189. 10.1371/journal.pone.0055189 PMC3572110 23418435

[42] Yang, X, Ge, C, Hu, B. Relationship between quality of life and occupational stress among teachers. Public Health. 20090;123:750–5. 10.1016/j.puhe.2009.09.018. 10.1016/j.puhe.2009.09.018 19883926

[43] Ministry of Health, Labour and Welfare 2015. https://www.mhlw.go.jp/bunya/roudoukijun/anzeneisei12/. Accessed 10 Mar 2021.

[44] ESG Investing: ESG Ratings—MSCI. https://www.msci.com/our-solutions/esg-investing/esg-ratings. Accessed 10 Mar 2021.

[45] Wooldridge JM (2010). Econometric analysis of cross section and panel data.

[46] Fu R, Noguchi H, Tachikawa H, Aiba M, Nakamine S, Kawamura A (2017). Relation between social network and psychological distress among middle-aged adults in Japan: evidence from a national longitudinal survey. Soc Sci Med.

[47] Ma X, Piao X, Oshio T (2020). Impact of social participation on health among middle-aged and elderly adults: evidence from longitudinal survey data in China. BMC Public Health.

[48] Piao X (2021). Marriage stability and private versus shared expenditures within families: evidence from Japanese families. Soc Indic Res.

